# An Amalgamated Approach to Bilevel Feature Selection Techniques Utilizing Soft Computing Methods for Classifying Colon Cancer

**DOI:** 10.1155/2020/8427574

**Published:** 2020-10-13

**Authors:** Sunil Kumar Prabhakar, Harikumar Rajaguru, Sun-Hee Kim

**Affiliations:** ^1^Department of Brain and Cognitive Engineering, Korea University, Anam-dong, Seongbuk-gu, Seoul 02841, Republic of Korea; ^2^Department of Electronics and Communication Engineering, Bannari Amman Institute of Technology, Sathyamangalam, India

## Abstract

One of the deadliest diseases which affects the large intestine is colon cancer. Older adults are typically affected by colon cancer though it can happen at any age. It generally starts as small benign growth of cells that forms on the inside of the colon, and later, it develops into cancer. Due to the propagation of somatic alterations that affects the gene expression, colon cancer is caused. A standardized format for assessing the expression levels of thousands of genes is provided by the DNA microarray technology. The tumors of various anatomical regions can be distinguished by the patterns of gene expression in microarray technology. As the microarray data is too huge to process due to the curse of dimensionality problem, an amalgamated approach of utilizing bilevel feature selection techniques is proposed in this paper. In the first level, the genes or the features are dimensionally reduced with the help of Multivariate Minimum Redundancy–Maximum Relevance (MRMR) technique. Then, in the second level, six optimization techniques are utilized in this work for selecting the best genes or features before proceeding to classification process. The optimization techniques considered in this work are Invasive Weed Optimization (IWO), Teaching Learning-Based Optimization (TLBO), League Championship Optimization (LCO), Beetle Antennae Search Optimization (BASO), Crow Search Optimization (CSO), and Fruit Fly Optimization (FFO). Finally, it is classified with five suitable classifiers, and the best results show when IWO is utilized with MRMR, and then classified with Quadratic Discriminant Analysis (QDA), a classification accuracy of 99.16% is obtained.

## 1. Introduction

A cancer is nothing but the abnormal growth of cells in the affected region, and it has the ability to spread to various regions of the body [1]. Colon cancer is one of the commonly occurring cancers, and it happens due to genetic, lifestyle, and aging factors. Other risk factors associated with it are lack of physical activity, obesity, diet issues, and smoking [2]. The main symptoms include blood in the stool, weight loss, fatigueness, and changes in the bowel movements. Often started as a benign tumor in the form of a polyp, later it becomes cancerous [3]. Treatments for colon cancer include radiation therapy, targeted therapy, chemotherapy, and surgery. The cancer may be cured if it is confined within the walls of the colon, but if it has spread widely, then it is not curable, but managed to a certain extent with improvement in life style quality [4].

For the identification of cancer disease, the microarray data classification technique is utilized widely [5]. To monitor genome wide expression, one of the vital tool that many biologists use is microarray technology. In the form of gene expression differences, the formulation and acquisition of data from tissue samples are obtained. Generally, huge size of scientific data brings a lot of problems to the researchers who are trying to identify the useful information for the application of data mining techniques to be used [6]. This tremendous amount of microarray data is also quite asymmetric in nature, as the number of genes ranges from a few hundreds to many thousands [7]. So, classification with this huge amount of data is difficult as it increases computational cost thereby degrades the performance of the classifier. Therefore, for such asymmetric data, it is very difficult to utilize the traditional classifiers, and therefore, for the analysis of microarray data, dimensionality reduction is highly required. A rank-based approach is mostly utilized to select the dominant features in the high dimensional data analysis [8]. Some of the common ranking approaches used in literature are Information gain,*t*-test, ANOVA, Relief F, BW ratio,*t*-statistic, Fischer score, correlation-based feature selection, Wilcoxon score test, Wilk's Lambda score, and Signal to Noise Ratio (SNR) Euclidean distance [9]. In this work, multivariate MRMR is used to select the top 600 genes. Later, with the optimization of using 6 techniques, the best 30, 60, and 90 genes are selected. Generally, the main intention of feature selection is multifarious as the comprehensibility of the classifier model mitigates, the unbalanced number of features and sample proportion reduces. For microarray-based classification of colon cancer, a few famous works reported in literature is given below.

A feature selection from colon cancer dataset for cancer classification using Artificial Neural Networks (ANN) was done by Rahman and Muniyandi [10]. The gene expression analysis was used to find out the risk analysis of colorectal cancer incidence by Shangkuan et al. [11]. Based on machine learning and similarity measures, gene selection and classification of colon cancer microarray data were done by Liu et al. [12]. Using multiple machine learning paradigms, the statistical characterization and classification of colon microarray gene expression data were done by Maniruzzaman et al. [13]. The prediction of colon cancer with genetic profiles utilizing intelligent technique was done by Alladi et al. [14]. For the diagnosis and survival prediction of colon cancer, ANN was proposed by Ahmed [15]. The polygon models for grandular structures [16] and the detection and classification of nuclei in routine colon cancer histology images were done by Sirinukunwattana et al. [17]. A deep learning-based tissue analysis prediction outcome in colorectal cancer was done by Bychkov et al. [18]. The colon cancer classification analysis using machine learning in DNA microarray data was used by Cho and Won [19]. An evolutionary neural network was utilized to predict the colon cancer by Kim and Cho [20]. A classification framework applied to cancer gene expression profiles was done by Hijazi and Chan [21]. A hybrid gene selection algorithm based on interaction information technology was utilized for microarray-based colon cancer classification [22]. A gene selection methodology based on clustering for classification tasks in colon cancer was done by Garzon and Gonzalez [23]. A hybrid gene selection method using MRMR and Artificial Bee Colony (ABC) was utilized for colon cancer classification by Alshamlan et al. [24]. A random subspace aggregation for colon cancer prediction was done by Yang et al. [25]. A supervised locally linear embedding technique with correlation coefficient was utilized for colon cancer microarray classification by Xu et al. [26]. Genetic programming was used for colon cancer classification by Vanneschi et al. [27]. Sparse representation for classification of colon tumor was done by Hang et al. [28]. A standardized comparative analysis of biomarker selection techniques was done by Dessi et al. [29]. A Node Influenced Method (NIM) for colon cancer classification was also used [30]. However, in this work, multivariate MRMR with six optimization techniques is used. The organization of the work is as follows. In Section 2, the materials and methods are given followed by the usage of MRMR technique to select the genes. In Section 3, the second level optimization using different optimization algorithms is done, and in Section 4, classifiers are explained followed by results in discussion in Section 5 and concluded in Section 6.

## 2. Materials and Methods

For the colon cancer classification, a dataset was used which is publicly available online [31]. There are about 2000 genes here. Class 1 represents the tumor class with 40 samples, and Class 2 represents the healthy class with 22 samples, and totally, there are 62 samples. The details of the dataset are tabulated in Table 1

**Figure 1:**
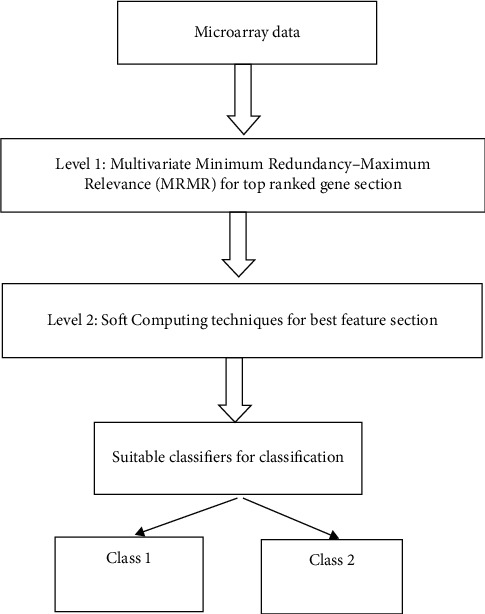
Illustration of the work.

The illustration of the work is shown in Figure 1.

### 2.1. Techniques to Select the Genes

The gene selection techniques utilized in this work are MRMR. The main aim of this work is to shortlist, rank, and extract the best 600 genes from 2000 genes.

#### 2.1.1. Multivariate Minimum Redundancy–Maximum Relevance (MRMR)

This technique minimizes the redundancy in each class and maximizes the gene relevancy with the class label [32] and with the help of several statistical measures, it is done.

The information which a random variable gives about another random variable with respect to both the gene activity and class label can be assessed by the Mutual Information (MI). For both continuous and categorical variables, this method can be applied. For discrete variables, MI is utilized to seek genes that are not redundant *R* and are maximally relevant *T*with an assigned target label and expressed as

(1) $$\begin{array}{c} R=\frac{1}{{\left|N\right|}^{2}}\underset{i,j\in F}{\sum }MI\left(i,j\right), \end{array}$$

(2) $$\begin{array}{c} T=\frac{1}{\left|N\right|}\underset{i\in F}{\sum }MI\left(h,i\right), \end{array}$$

where *MI* represents Mutual Information, *i*, *j* represents the genes, |*F*| represents the number of features in *N*, and *h* represents the class label.

For continuous variables, the *F*-statistic (ANOVA test) is utilized to trace the maximum relevance between a gene and a class label. To minimize redundancy, the measurement of the correlation of the gene pair of that class is done as

(3) $$\begin{array}{c} T=\frac{1}{\left|N\right|}\underset{i\in N}{\sum }F\left(i,h\right), \end{array}$$

(4) $$\begin{array}{c} R=\frac{1}{{\left|N\right|}^{2}}\underset{i,j\in N}{\sum }\left|c\left(i,j\right)\right|, \end{array}$$

where the *F*-statistic is expressed as *F*, *i*, *j* are the genes, and the class label is represented as *h*. The number of factors in *N* is |*N*|; *c* represents correlation. It is utilized together with entropy. To analyze the relevance and redundancy of a gene cluster, then normalized MI is utilized, and the combination of the most relevant genes is traced. For continuous variables, linear relationships are replaced by MI. For both discrete and categorical data, this method gives lower error accuracies.

## 3. Optimization Techniques

The solution of the best element from a particular set of available alternatives can be done with the help of optimization techniques [33]. Application ranging from computer science, economics, biology, mechatronics, etc. has utilized optimization techniques predominantly based on their necessity. Therefore, optimization is nothing, but the minimization of a real function by means of choosing the input values systematically from a specific set and then the value of the functions is computed. Therefore, in a defined domain, to find the best available values of a particular objective function, optimization is used. This work utilizes the usage of 6 optimization techniques to find the best values of 30, 60, and 90 features/genes from the 600 shortlisted genes so that it can be well classified, thus forming a very nice, amalgamated approach

### 3.1. Invasive Weed Optimization

A famous population based meta heuristic algorithm is IWO [34]. By utilizing the randomness and imitating property of weeds colony, the general optimum of a mathematical function is found out. A serious threat to crops is the growth of weeds as they have an offensive growth habit. They are very powerful as they are quite adaptable and resistant to environmental changes. A powerful and simple optimization algorithm is obtained when their characteristics are considered. Initiation of three different qualities of a weed such as randomness, resistance, and adaptability is considered by this algorithm. In agriculture, this technique is inspired by the methodology having colonies of invasive weeds. A weed is nothing but a plant which grows all of a sudden and unintentionally; though when weeds grow in other places where it does not interfere with the basic human needs, then it is not considered as a problem. Based on the colonized weed, a simple numerical optimization algorithm has been proposed, and it is called as IWO algorithm. This algorithm is very powerful and effective in optimal solutions convergence with the help of utilizing preliminary features such as seeding, growth of it, and competition in a weed colony. Some basic features by the method to simulate the habitat behavior of weeds are considered as follows:

1. Initialization of primary populations: in the search space, the distribution of limited number of seeds is done
2. Process of reproduction: a flowering plant is obtained from each seed, and again, flowering plant pushes seeds based on their fitness value. In a linear manner, there is a decrease in the number of grains of grasses from *A*_max_ to *A*_min_ as follows:

(5) $$\begin{array}{c} n\left({\text{weed}}_{j}\right)=\frac{A_{\max}\left(\max\text{fit}-\text{fit}\left({\text{weed}}_{j}\right)\right)+A_{\min}\left(\text{fit}\left({\text{weed}}_{j}\right)-\min\text{fit}\right)}{\max\text{fit}-\min\text{fit}}. \end{array}$$

(3) Spectral Spread Method: the seeds obtained by the group are represented in a normal distribution with a mean standard deviation and is expressed as

(6) $$\begin{array}{c} \sigma_{t}={\left(\frac{T-t}{T}\right)}^{m}\left(\sigma_{\text{initial}}-\sigma_{\text{final}}\right)+\sigma_{\text{final}}, \end{array}$$

where the number of maximum iterations is represented as *T*, the current standard deviation is *σ*_*t*_, and the nonlinear modulation index is represented as *m*.

This equation convinces that in a nonlinearly manner, there is a decrease of the fall of grains so that more fit plants are produced, and inappropriate plants are eliminated.

(4) Competitive deprivation: if the number of grasses becomes higher than the maximum number of grasses in the colony (*C*_max_), the grass having the worst fitness value is removed from the colony so that in the colony, a standard number of herbs remains.
(5) Until the maximum number of iterations are reached, this process continues, and then, storage of minimum colony cost function of the grasses is done.

### 3.2. Teaching Learning-Based Optimization

One of the famous population-based optimization techniques is TLBO where a classic teaching-learning phenomenon is mimicked within a particular classroom environment [35]. Here, a group of learners is assumed as population, and various design variables are assumed as different subjects provided to the learners. Therefore, the learner's results are highly analogous to the fitness value of the optimization problem. The last solution in the entire population is assumed as the teacher. Teacher phase and learner phase are the two important phases of TLBO, and the two phases are elaborated as follows:

#### 3.2.1. Teacher's Phase

In this stage, the learning is done by the learners from the teachers. The enhancement of the mean of the whole class to the learner's level is tried by the teacher in this phase. Between the existing mean and the new mean, the difference is expressed as

(7) $$\begin{array}{c} \text{Diff}\_{\text{Mean}}_{j}=n_{j}\left(E_{\text{new}}-T_{F}E_{j}\right), \end{array}$$

where *E*_new_ represents the new mean for the *j*^th^ iteration and*E*_*j*_ represents the mean for each design variable.

Two randomly generated parameters are applied within the equation: *n*_*j*_ is the range number between 0 and 1. The teaching factor is represented as *T*_*F*_, and here, in our work, it is set as 2. By setting the value as 2, it has a major effect on the value of the mean to be changed. The role of the adjusting factor is played by *T*_*F*_ in this algorithm which helps to control the scale and moving direction when the solutions are updated. In a random manner, the value of *T*_*F*_ is decided and is represented as

(8) $$\begin{array}{c} T_{F}=\text{round}\left\{1+\mathrm{rand}\left(0,1\right)\left\{2-1\right\}\right\}. \end{array}$$

The existing solution is updated based on this Diff_Mean according to the following expression as:

(9) $$\begin{array}{c} A_{\text{new},j}=A_{\text{old},j}+\text{Diff}\_{\text{Mean}}_{j}. \end{array}$$

#### 3.2.2. Learner's Phase

In this second part of the algorithm, the learners interact between themselves and increase their knowledge. Random interaction between one learner and the other learner occurs so that the knowledge is enhanced. If a particular learner has more knowledge, then other learners can make use of this learner with good knowledge and can improve their skills. The learning phenomenon is expressed mathematically as follows:

At a specific iteration *j*, *A*_*j*_, and *A*_*k*_ are considered as two different learners (solutions), where *j* ≠ *k* and is represented as

(10) $$\begin{array}{c} A_{\text{new},j}=A_{\text{old},j}+n_{j}\left(A_{k}-A_{j}\right)\text{if}\;f\left(A_{k}\right)<f\left(A_{j}\right) \end{array}$$

(11) $$\begin{array}{c} A_{\text{new},j}=A_{\text{old},j}+n_{j}\left(A_{j}-A_{k}\right)\text{if }f\left(A_{j}\right)<f\left(A_{k}\right). \end{array}$$

If *A*_new_ provides a better function value, then it is accepted into the population. For the implementation of TLBO, the steps are as follows:

**Step 1 .**

The optimization problem is defined, and the algorithm parameters are initialized. The population size (*P*_*s*_) is initialized along with the total number of generations (*G*_*s*_) and the number of design variables (*D*_*s*_). The optimization problem is defined as follows for our case: minimize *f*(*A*), where *f*(*A*) is the objective function and *A* denotes the vector for design variables. Initial solutions are constructed as per *P*_*s*_ and *D*_*s*_.

**Step 2 .**

The mean of the population columnwise is calculated so that the mean of each degree variable is obtained as *E*_*j*_. The best solution as (teacher) is identified based on *A*_teacher_ = *A*_*f*(*A*)=min_; the movement of *E*_*j*_ to *A*_teacher_ will be tried hard, so assume *E*_new_ = *A*_teacher_.

**Step 3 .**

The diff_mean based on (8) is calculated by using the teacher factor *T*_*F*_ effectively.

**Step 4 .**

Based on (9), the solution in the teacher phase is modified, and the new solution is accepted if it is better than the existing one.

**Step 5 .**

Based on (10) and (11), the solution in the learner phase is updated, and then, the better one is accepted into population.

**Step 6 .**

Until the termination criterion is met, the steps (2) to (5) are repeated.

### 3.3. League Championship Optimization

LCO is a new evolutionary algorithm inspired from sporting competitions in various sports leagues, and its main intention over a continuous search space is tracing the optimum solution for problems done here [36].

A randomly created group of ′*A*′ solutions forms the initial population of the algorithm. To a team, all the solutions are being attributed specifically to the formation of the current team. The playing strength that is being assigned has the fitness value, and it is very useful for the formation of corresponding team. A more potent formation is aimed to replace the present formation, and it is because of the greedy selection of the LCO. The number of seasons (*N*) is assigned as a termination factor which compresses *A* − 1 weeks so that *N* × (*A* − 1) contest weeks are yielded (it is to be understood that *A* is an even value).

The existing teams always play in pass when the league schedule in every week is considered. Depending on the team formation, the playing strength of the team assesses the match outcome. When the events of previous events are tracked, during the recovery time, the formation and update of each team is done. The famous rule of the LCO is that if the value of playing strength is more then the likelihood of winning the game is more, the prediction about the outcome of a match cannot be done, and also win/lose could only be represented.

1. *League Schedule Development*. For a season, the generation of a nonrandom order is done to enable teams so that a match is played against each other. By making a single round robin program, LCO does this task so that during a season, only one match is held between 2 teams. When the involvement of ′*A*′ teams is done, then *A*(*A* − 1) (2 games are required).
2. *Winner/loser Determination*. As per the standard rule (the higher probability of winning if the playing strength of a team is higher) and considering *Z*_*j*_^*w*^ and *Z*_*k*_^*w*^ as the formations and also *f*(*Z*_*j*_^*w*^) and *f*(*Z*_*k*_^*w*^) as the playing strength of the teams *j* and *k*, then

(12) $$\begin{array}{c} \frac{f\left(Z_{j}^{w}\right)-\overset{⌢}{f}}{f\left(Z_{k}^{w}\right)-\overset{⌢}{f}}=\frac{C_{k}^{w}}{C_{j}^{w}}, \end{array}$$

where *C*_*k*_^*w*^ is the chance of a particular team ′*k*′ to its opponent at week *w*, (*C*_*j*_^*w*^)is defined accordingly and *f*[*Z* = (*z*_1_, *z*_2_, .., *z*_*M*_)] is an *M* variable function which is aimed to be reduced over the entire space.

The above mentioned formula indicates that the likelihood probability of a win for the particular team *k* (or *j*) is highly proportional to the difference between *f*(*Z*_*k*_^*w*^) or *f*(*Z*_*j*_^*w*^) and the total strength of the team. In such a case, a better team is assumed to have more factors in compliance with the ideal team. For the evaluation of the team, the distance from a common reference point forms as the basis. Therefore, for the winning portion of team is expressed by the ratio of these distances. When the idealized rule is considered, from the viewpoint of both teams, the probability that team *j* beats team *k* is considered to be equal and is expressed as

(13) $$\begin{array}{c} C_{k}^{w}+C_{j}^{w}=1. \end{array}$$

From (12) and (13), *C*_*j*_^*w*^ is expressed as follows

(14) $$\begin{array}{c} C_{j}^{w}=\frac{f\left(Z_{k}^{w}\right)-\hat{f}}{f\left(Z_{k}^{w}\right)+f\left(Z_{j}^{w}\right)-2\hat{f}}. \end{array}$$

Then, random generation of a number considered from 0 to 1 is done, and it is compacted with *C*_*j*_^*w*^ to assess the winning/losing team. So team ′*j*′ won the game if *C*_*j*_^*w*^ is greater than or equal to this, if not otherwise vice-versa happens.

### 3.4. Beetle Antennae Search Optimization

The richest species of the order Coleoptera is beetles. There are two long antennae in the beetles, and they are usually longer than the body. For detecting the food resources and a potential suitable mate, the two antennae can be utilized. When the unknown areas are explored, these antennae can act as an exploration apparatus. A metaheuristic algorithm can be modelled using the exploration behavior of beetles with two antennae, and it is called BAS algorithm [37]. The achievable solution is represented by the position of every beetle, and so the optimal solution is considered as the least and minimum distance from food. Without the gradient information itself, the optimization of BAS can be done. The particular search process is explained as follows:

**Step 7 .**

All the BAS algorithm parameters are defined. The initialization of *P* positions of beetles *x*_*p*_(*p* = 1, 2, ⋯, *P*) is done randomly. The maximum number of iterations is set as *I*_max_ and set *i* = 0.

**Step 8 .**

In a random dimensional space, the initial antennae positions of beetles are constructed to be normalized so that the initial exploration environment can be expanded. The normalization of a random searching direction is done as follows:

(15) $$\begin{array}{c} \vec{q}=\left(\frac{\text{rnd}\left(\dim,1\right)}{\left\|\text{rnd}\left(\dim,1\right)\right\|}\right), \end{array}$$

where a random function is denoted as rnd(.) whose dimension of the solution is represented as dim.

**Step 9 .**

To assess the location of food, beetles utilize their antennae when foraging. If the antenna on one particular side is close to food, then the odor of food is received by that antenna, and as a result, it becomes stronger thereby the individual progresses to that same antenna side. The normalization of the right and left antennae is done as follows:

(16) $$\begin{array}{c} z_{r}^{i}=z^{i}+s^{i}.\vec{q}, \end{array}$$

(17) $$\begin{array}{c} z_{l}^{i}=z^{i}-s^{i}.\vec{q}, \end{array}$$

where the iteration number is represented as *i*; the position of the left and right antennae is represented as *z*_*r*_^*i*^ and *z*_*l*_^*i*^, respectively. The position of the beetle is represented as *z*^*i*^, and the sensing length of the antennae is represented as *s*^*i*^.

**Step 10 .**

By means of detecting the odor, the determination of the next position of beetle is done. So, based on the strength of the odor, the next location of the beetle is explored. Whichever antenna (left or right) receives the strongest odor, then the progress or movement will be towards it. The update of the beetle's location is done as:

(18) $$\begin{array}{c} z^{i+1}=z^{i}+m.\delta^{i}.\vec{q}.\mathrm{sign}\left(f\left(z_{r}^{i}\right)-f\left(z_{l}^{i}\right)\right), \end{array}$$

where the step size of searching is represented as *δ*_*i*_, *f*(.) is represented as the evaluation function, and *m* represents the movement direction of the beetle. The sign function is represented as sign (.)

(19) $$\begin{array}{c} \mathrm{sign}\left(f\left(z_{r}^{i}\right)-f\left(z_{l}^{i}\right)\right)=\left\{\begin{array}{c} 1\begin{array}{cc} & f\left(z_{r}^{i}\right)-f\left(z_{l}^{i}\right)>0, \end{array}\\ 0\begin{array}{cc} & f\left(z_{r}^{i}\right)-f\left(z_{l}^{i}\right)=0, \end{array}\\ -1\begin{array}{cc} & f\left(z_{r}^{i}\right)-f\left(z_{l}^{i}\right)<0. \end{array} \end{array}\right. \end{array}$$

**Step 11 .**

The update of the sensing length of the antenna *s*^*i*^ and the searching step size *δ*^*i*^ is done as follows:

(20) $$\begin{array}{c} s^{i+1}=c_{1}*s^{i}+0.01, \end{array}$$

(21) $$\begin{array}{c} \delta^{i+1}=c_{2}*\delta^{i}. \end{array}$$

The fixed reduction factors are represented as *c*_1_ and *c*_2_ (between 0 and 1).

**Step 12 .**

The evaluation function of every individual is computed and compared to all the possible solutions to assess the optimal solutions. The number of iterations is updated as *i* = *i* + 1 and returns to step 2. Until *i* = *I*_max_is achieved, the process is repeated.

**Step 13 .**

The optimal solution is expressed as output.

### 3.5. Crow Search Optimization

A famous metaheuristic algorithm, its application is widely used in many fields/problems. It is basically inspired from the highly intelligent attitude and behavior of crows [38]. Naturally, intelligence behaviors can be well seen in crows such as self-awareness, recognizing faces, advanced communication ways between them, warning the flock between unfriendly ones, and recognition of the food's hidden place after a long period of term. When compared to the human brain, the brain body ration of the crows is slightly lower, and crows in general have been recognized as the one of the most intelligent birds in nature.

The natural behavior of crows is emulated by the CSO evolutionary process by means of hiding and recovering the food. This algorithm is primarily based on population, and so the flock size is confirmed by *M* crows or individuals which are of *m*-dimensional in nature. The position on *Y*_*j*,*g*_ of the crow *j* in a particular iteration *g* is expressed and indicated as a possible solution represented as

(22) $$\begin{array}{c} Y_{j,g}=\left[y_{j,g}^{1},y_{j,g}^{2},\cdots ,y_{j,g}^{m}\right];j=1,2,\cdots ,M, \end{array}$$

(23) $$\begin{array}{c} g=1,2,\cdots \max\text{iter}, \end{array}$$

where the maximum of iterations in this method is expressed as maxiter. The best visited location *L*_*j*,*g*_ is remembered by the crow due to its natural capability in order to hide food until the current iterations are expressed as:

(24) $$\begin{array}{c} L_{j,g}=\left[l_{j,g}^{1},l_{j,g}^{2},\cdots ,l_{j,g}^{m}\right]. \end{array}$$

Based on two behaviors, pursuit and evasions, the modifications of each position, are done as follows:

1. Pursuit: with the main intention to discover the hidden place, a crow ′*k*′ follows crow ′*j*′. The purpose of crow ′*k*′ is achieved, and the crow *j* does not check the presence of other crow
2. Evasion: the crow ′*j*′ deliberately takes a random trajectory in order to protect its food as the crow ′*j*′ knows about the presence of crow ′*k*′. By implementing a random movement, the simulation of the behavior in CSO is done

An Awareness Problem (AP) is met to determine the kind of behavior considered by each crow ′*j*′. Therefore, a uniform distribution of a random value *r*_*j*_ between the ranges of 0 and 1 is sampled. If the range *r*_*j*_ is greater or equal than AP, then application of behavior is implemented, or else situation two is chosen. In the following model, the summarization is done as:

(25) $$\begin{array}{c} Y_{j,g+1}=\left\{\begin{array}{c} Y_{j,g}+r_{j}.{\text{fl}}_{j,g}.\left(L_{k,g}-Y_{j,q}\right)\begin{array}{cc} & r_{j}\geq \text{AP} \end{array}\\ \text{random}\begin{array}{cc} & \begin{array}{cc} & \begin{array}{cc} & \text{otherwise}. \end{array} \end{array} \end{array} \end{array}\right. \end{array}$$

The magnitude of movement from crow *Y*_*j*,*g*_ towards the best position *L*_*k*,*g*_ of crow *k* is indicated by the flight length *fl*_*j*,*g*_. The random number *r*_*j*_ is in the range of [0, 1] with uniform distribution. The evaluation of their position and the update of the memory vector are done as follows once the modification of crows is done as follows:

(26) $$\begin{array}{c} L_{j,g+1}=\left\{\begin{array}{c} F\left(Y_{j,g+1}\right)\begin{array}{cc} & F\left(Y_{j,g+1}\right)<F\left(L_{j,g}\right) \end{array}\\ L_{j,g}\begin{array}{cc} & \begin{array}{cc} & \begin{array}{cc} & \begin{array}{cc} & \text{otherwise}, \end{array} \end{array} \end{array} \end{array} \end{array}\right.. \end{array}$$

where the objective function to be minimized is represented as *F*(.).

### 3.6. Fruit Fly Optimization

A famous relatively fast and simple method to find global optimization is FFO algorithm, and it is dependent on the food finding behavior of the fruit fly [39]. The smell of the food source can attract the fruit fly even when it is at a faraway location, and then, it progresses towards that direction rapidly. Once it gets close to the location of the food, it utilizes its vision to trace the food. FFO when compared with other optimization algorithms can achieve accurate optimization quickly.

The summary of the original FFO is as follows:

**Step 14 .**

Initialization: for the fly group, the population size is defined with the random initial fruit fly swarm location (*X*_axis, *Y*_axis) and the iteration termination criteria.

**Step 15 .**

Specific location assignment: the fruit fly location (*A*_*i*_, *B*_*i*_) of an individual is randomly assigned as

(27) $$\begin{array}{c} A_{i}=X\_\text{axis}+\begin{array}{cc} \text{Random} & \text{Value}, \end{array} \end{array}$$

(28) $$\begin{array}{c} B_{i}=Y\_\text{axis}+\begin{array}{cc} \text{Random} & \text{Value} \end{array}. \end{array}$$

**Step 16 .**

The smell concentration judgement value *SC*_*i*_ is set as the reciprocal of the distance from the fruit fly to the origin as

(29) $$\begin{array}{c} \text{Distance}:{\text{SC}}_{i}=\frac{1}{\text{Distance}}. \end{array}$$

**Step 17 .**

The smell concentration judgement function is defined, and it is nothing but the fitness function. For the corresponding position, it is done by substituting *SC*_*i*_ to trace the smell concentration.

**Step 18 .**

The maximum smell concentration value along with its corresponding position is found out:

(30) $$\begin{array}{c} \left[\text{bestSmell},\text{bestSmell}\right]=\max\;\left(\text{Smell}\right). \end{array}$$

**Step 19 .**

The maximum smell location is utilized to replace the swarm centre location and is represented as:

(31) $$\begin{array}{c} \text{Smellbest}=\text{bestSmell}, \end{array}$$

(32) $$\begin{array}{c} X\_\text{axis}=A\left(\text{bestIndex}\right), \end{array}$$

(33) $$\begin{array}{c} Y\_\text{axis}=Y\left(\text{bestIndex}\right). \end{array}$$

**Step 20 .**

The swarm history best Smellbest should be superior to bestSmell so that it is proceeded to step 6. Otherwise, proceed to step 2 and continue the iteration.

## 4. Classification Procedures

The best selected feature values or the optimized values are then used for classification. Five different types of classifiers are used here in this work.

### 4.1. Random Forest (RF) Classifiers

One of the famous ensemble learning technique for regression and classification is Random Forest. With the help of bootstrap aggregation, multiple Decision Trees are constructed here. Based on the prediction of the tree structure, the classification is done by RF. After attaining the ultimate solution in the majority voting system, the judging of the result of each tree is done, and so it is highly suitable for a better fit.

### 4.2. Adaboost Classifiers

A famous machine learning technique is Adaboost meaning adaptive boosting. To improve the performance of the classifier, it is utilized in conjunction with various kinds of algorithm. This classifier is generally less prone to overfitting problems and quite sensitive to noisy data. To achieve an optimal classification performance on a dataset, many parameters should be adjusted based on the appropriate learning algorithm, and Adaboost does it so well.

### 4.3. Logistic Regression (LR)

It is a famous supervised learning classifier. When the input variable is either discrete or continuous and when the output variable is categorical, it is used widely. Based on the input variables, the parameters are estimated by the Logistic Regression so that the probability of output variable is exactly predicted.

### 4.4. Decision Trees (DT)

It is a famous decision support tool that utilizes a tree structure constructed using input features. Based on many input features, the target variables are easily predicted and that is the main objective of this classifier. Almost for different kinds of applications, DTs are used because for a given input data, the extraction of decision rules can be done easily.

### 4.5. Quadratic Discriminant Classifier (QDA)

A famous supervised learning technique in machine learning field, it is widely used by many researchers to classify the objects into 2 or more classes by means of using a quadratic surface. It is a simple extension of LDA, and the rule of classification is the same as it. Here, among the groups, equal covariance matrices are not assumed generally.

## 5. Results and Discussion

It is classified with a 10-fold cross validation method, and the performance of it is shown in tables below. The mathematical formulae for computing the Performance Index (PI), Sensitivity, Specificity, and Accuracy are mentioned in literature, and using the same, the values are computed and exhibited [33]. PC is Perfect Classification; MC is Missed Classification, and FA is False Alarm in the expressions below. In addition to that, Good Detection Rate (GDR) is also computed and shown.

The Sensitivity is computed as

(34) $$\begin{array}{c} \text{Sensitivity}=\frac{\text{PC}}{\text{PC}+\text{FA}}\times 100. \end{array}$$

Specificity is computed as

(35) $$\begin{array}{c} \text{Specificity}=\frac{\text{PC}}{\text{PC}+\text{MC}}\times 100. \end{array}$$

Accuracy is expressed as

(36) $$\begin{array}{c} \text{Accuracy}=\frac{\text{Sensitivity}+\text{Specificity}}{2}. \end{array}$$

Performance Index (PI) is expressed as

(37) $$\begin{array}{c} \text{PI}=\left(\frac{\text{PC}‐\text{MC}‐\text{FA}}{\text{PC}}\right)\times 100. \end{array}$$

Good Detection Rate (GDR) is calculated as

(38) $$\begin{array}{c} \text{GDR}=\left(\frac{\left[\text{PC}‐\text{MC}\right]}{\left[\text{PC}+\text{FA}\right]}\right)\times 100. \end{array}$$

Table 2 shows the performance analysis of classifiers in terms of classification accuracies with six optimization techniques for different gene selection methods using 30-60-90 selected genes. It is revealed from Table 2 that QDA classifier with 90 selected genes at IWO technique reached the highest accuracy of 99.16%. LR classifier with 60 selected genes attained a lower value of classification accuracy of 75.609% at CSO under individual category. Across the classifiers, the FFO method acquired a high average accuracy of 91.43%.

Table 3 demonstrates the performance analysis of classifiers in terms of PC with six optimization techniques for different gene selection methods using 30-60-90 selected genes. It is observed from Table 3 that QDA classifier with 90 selected genes at IWO technique reached the highest PC of 98.96%. Adaboost classifier with 30 selected genes attained a lower value of PC of 51.125% in CSO under individual category. Across the classifiers, the FFO method maintained a high average PC of 82.865%. This is due to the smoothening effect of features by FFO across the classifiers.

Table 4 reports the performance analysis of classifiers in terms of PI with six optimization techniques for different gene selection methods using 30-60-90 selected genes. From Table 4, it is observed that QDA classifier with 90 selected genes at IWO technique reached the highest PI of 98.935%. Adaboost classifier with 30 selected genes ebbed at a lower value of PI of 4.391% in CSO under individual category. Across the classifiers, the FFO method maintained high average PI of 76.672%. The lowest ever average PI of 29.83% across the classifiers is indicated by BASO method.

Table 5 depicts the performance analysis of classifiers in terms of GDR with six optimization techniques for different gene selection methods using 30-60-90 selected genes. From Table 5, it is reported that QDA classifier with 90 selected genes at IWO technique reached the highest GDR of 98.96%. LR classifier with 60 selected genes was ebbed at a lower value of GDR of 4.758% in CSO under individual category. Across the classifiers, the FFO method maintained high average GDR of 82.86%. The lowest average GDR value of 53.16% across the classifiers is attained by the BASO method.

Table 6 deals with the average performance analysis of classifiers in terms of parameters like accuracy, PC, PI, and GDR in average to six optimization techniques for different gene selection methods using 30-60–90 selected genes. It is indicated in Table 6 that QDA classifier with 90 gene selected condition scores higher parametric values like accuracy of 91.22%, PC of 82.36%, PI of 72.44%, and GDR of 82.37%. Therefore, QDA classifier with 90 gene selected gene cases will be considered as a better performing classifier than others. The RF classifier with 90 gene selected condition arrived low values for bench mark parameters like accuracy of 82.42%, PC of 64.85%, and PI of 40.919%, respectively. The lower value of 46.317% of GDR parameter is steeled by LR classifier with 60 gene selected condition.

**Figure 2:**
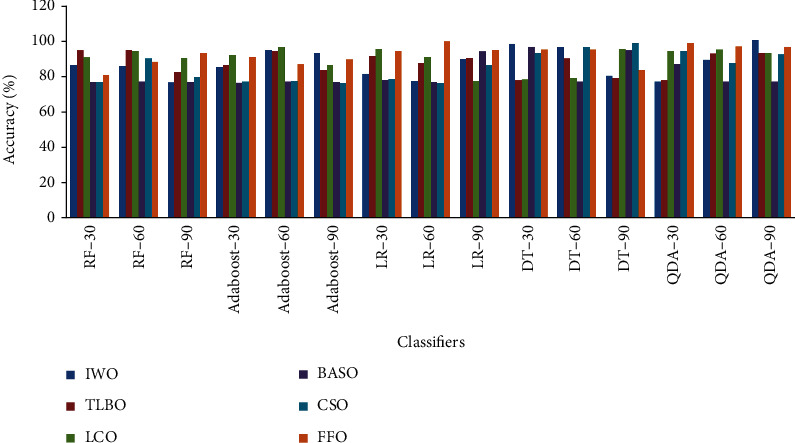
Performance analysis of accuracy in various classifiers under six different optimization methods for 30-60-90 gene selected in colon cancer.

Figure 2 shows the performance analysis of accuracy in various classifiers under six different optimization methods for 30-60-90 genes selected in colon cancer. As depicted from Figure 2 that QDA classifier with 90 selected genes at IWO technique reached the highest accuracy of 99.16%. LR classifier with 60 selected genes attained a lower value of classification accuracy of 75.609% at CSO. Across the classifiers, the FFO method acquired a high average accuracy of 91.43%. The lower average accuracy of 80.55% is ebbed by the BASO method.

**Figure 3:**
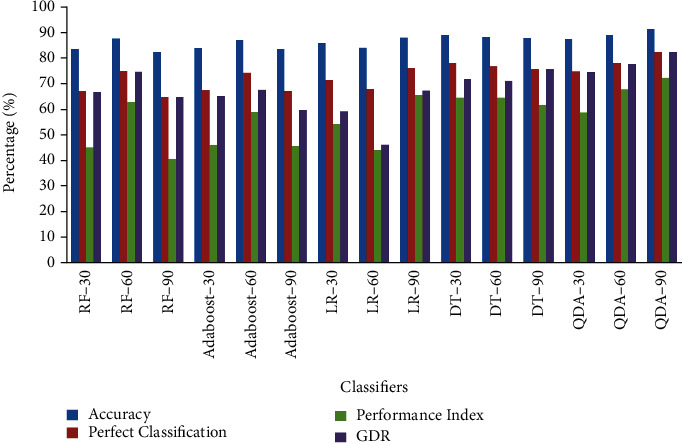
Average performance analysis of classifier parameters.

Figure 3 represents the average performance analysis of classifier bench mark parameters like accuracy, PC, PI, and GDR. It is demonstrated from Figure 3 that QDA classifier with 90 gene selected condition scores higher parametric values like accuracy of 91.22%, PC of 82.36%, PI of 72.44%, and GDR of 82.37%. Therefore, QDA classifier with 90 gene selected cases will be considered as a better performing classifier than others. The RF classifier with 90 gene selected conditions arrived low values of parameters like accuracy of 82.42%, PC of 64.85%, and PI of 40.919%, respectively. The lower value of 46.317% of GDR parameter is maintained by LR classifier with 60 gene selected condition.

## 6. Conclusion and Future Work

Thus, the classification of colon cancer has a huge importance in the medical field. As many existing cancer classification models are clinical based, it has a pretty less diagnostic ability. With the rapid advancement of gene expression technology, many kinds of cancers can be classified with the help of using DNA microarray. As the characteristics of gene expression data possess a high dimension, nonbalanced distribution, and a small sample size, classification of it is pretty difficult. Therefore, to get a better insight into the colon cancer classification problem, a systematic approach has been proposed. In this paper, the problem of colon cancer classification is confronted with the help of MRMR and six other optimization techniques. Finally, it is classified with five suitable classifiers, and the best results show when IWO is utilized with MRMR, and then classified with QDA, a classification accuracy of 99.16% is obtained. Future works aim to work with other feature selection techniques and optimization methods for the better classification and analysis of microarray-based colon cancer classification.

## Figures and Tables

**Table 1:** Dataset details.

Dataset	Number of genes	Class 1 (tumor)	Class 2 (healthy)	Total samples
Colon cancer	2000	40	22	62

**Table 2:** Performance analysis of classifiers in terms of classification accuracies with six optimization techniques for different gene selection methods using 30-60-90 selected genes.

Classifiers	Gene selection	Optimization techniques					
		Invasive Weed Optimization	Teaching Learning-Based Optimization	League Championship Optimization	Beetle Antennae Search Optimization	Crow Search Optimization	Fruit fly Optimization
RF	30	85.74344	93.75	89.85625	76.135	76.135	79.94629
	60	85.22406	94.01125	93.36	76.3375	89.20625	87.48398
	90	75.84375	81.9	89.6	76	78.71281	92.51797

Adaboost	30	84.375	85.67875	91.1475	75.75	76.3375	89.96621
	60	94.01125	93.555	95.575	76.3375	76.75938	85.9375
	90	92.19	82.942	85.74344	76.23625	75.625	88.62695

LR	30	80.86	90.49688	94.53375	77.27594	77.83109	93.49766
	60	76.675	86.76016	90.1125	76.25313	75.60938	98.69688
	90	88.8125	89.6	76.86906	93.23	85.54938	94.03444

DT	30	97.395	77.05469	77.53719	95.705	92.19	94.23867
	60	95.575	89.20625	78.38625	76.3375	95.575	94.23867
	90	79.69	78.45156	94.795	93.75	97.655	82.86824

QDA	30	76.37125	77.08	93.555	86.32813	93.36	97.915
	60	88.55	91.93	94.2725	76.3375	86.66	95.835
	90	99.16	92.19	92.19	76.3375	91.47912	95.70313

Average		86.71975	86.97377	89.1689	80.55673	84.57899	91.43377

**Table 3:** Performance analysis of classifiers in terms of PC with six optimization techniques for different gene selection methods using 30-60–90 selected genes.

Classifiers	Gene selection	Optimization techniques					
		Invasive Weed Optimization	Teaching Learning-Based Optimization	League Championship Optimization	Beetle Antennae Search Optimization	Crow Search Optimization	Fruit fly Optimization
RF	30	71.48688	87.5	79.69	52.27	52.27	59.89258
	60	70.44813	88.0225	86.72	51.625	78.38813	74.96797
	90	51.6875	63.8	79.17	52	57.42563	85.02938

Adaboost	30	68.75	71.3575	82.295	51.5	51.125	79.93242
	60	88.0225	87.11	91.15	55.00906	53.51875	71.875
	90	84.38	65.884	71.48688	52.4725	51.25	77.24688

LR	30	61.72	80.99	89.0675	54.55188	55.66219	86.97938
	60	53.35	73.50344	80.21	52.50625	51.21875	97.39375
	90	77.60625	79.17	53.73813	86.46	71.09875	88.06888

DT	30	94.79	54.10938	55.07438	91.41	84.38	88.47734
	60	91.15	78.38813	56.7725	55.12336	91.15	88.47734
	90	59.38	56.90313	89.59	87.5	95.31	65.73648

QDA	30	52.7425	54.16	87.11	72.65625	86.72	95.83
	60	77.085	83.86	88.545	54.10938	73.31953	91.67
	90	98.96	84.38	84.38	52.135	82.95589	91.40625

Average		73.43725	73.94254	78.33329	61.42191	69.05284	82.86558

**Table 4:** Performance analysis of classifiers in terms of PI with six optimization techniques for different gene selection methods using 30-60-90 selected genes.

Classifiers	Gene selection	Optimization techniques					
		Invasive Weed Optimization	Teaching Learning-Based Optimization	League Championship Optimization	Beetle Antennae Search Optimization	Crow Search Optimization	Fruit fly Optimization
RF	30	60.105	85.7	78.4275	8.64875	8.64875	30.28875
	60	58.02563	86.37	84.66125	6.27625	76.62938	65.66844
	90	6.511875	43.225	78.93	7.69	25.80313	83.35969

Adaboost	30	54.54	59.85	78.465	5.805	4.39125	73.49922
	60	86.37	85.18063	90.78	18.14688	13.08297	60.87
	90	81.4325	48.1615	60.105	9.367813	4.8625	70.92234

LR	30	37.86063	77.17125	87.71	16.64625	20.29063	86.7675
	60	12.48375	63.30375	77.925	9.487656	4.744688	97.28125
	90	74.32875	78.93	13.86195	84.315	59.34	83.59028

DT	30	94.49	15.18023	18.36125	91.18	81.4325	87.07812
	60	90.78	76.62938	23.8125	18.52203	90.78	87.07812
	90	31.4425	24.21063	88.38	85.7	95.07	45.81047

QDA	30	10.32656	15.36	85.18063	62.3175	84.66125	95.65
	60	72.795	80.72125	87.04	15.18023	59.64609	91.58
	90	98.935	81.4325	81.4325	8.169375	74.04878	90.64688

Average		58.02848	61.42841	69.00484	29.83018	46.89546	76.67274

**Table 5:** Performance analysis of classifiers in terms of GDR with six optimization techniques for different gene selection methods using 30-60 – 90 selected genes.

Classifiers	Gene selection	Optimization techniques					
		Invasive Weed Optimization	Teaching Learning-Based Optimization	League Championship Optimization	Beetle Antennae Search Optimization	Crow Search Optimization	Fruit fly Optimization
RF	30	71.48527	85.71429	79.69	52.27	52.27	59.89258
	60	70.44469	86.39268	86.72	51.625	78.38813	74.96797
	90	51.6875	63.80399	79.17	52	57.42563	85.02938

Adaboost	30	54.54545	71.35536	82.295	51.5	51.125	79.93242
	60	88.0225	87.11	91.15	55.00906	13.1496	71.875
	90	84.38	65.88466	71.48527	9.423984	51.25	77.24688

LR	30	61.72	80.99	89.0675	16.68824	20.34483	86.98345
	60	12.55858	73.50344	80.21	9.546483	4.758999	97.32657
	90	71.1444	73.68953	13.91238	86.46	71.09555	88.07165

DT	30	94.79474	54.10938	18.42734	91.41	84.38	88.47734
	60	90.29073	78.38813	23.85838	55.12336	91.15	88.47734
	90	59.38	56.90313	89.59	87.5	95.31715	65.73803

QDA	30	52.7425	54.16	87.11	72.65625	84.68635	95.83958
	60	77.085	83.86	87.06308	54.10938	73.3201	91.67
	90	98.96	84.38	84.38	52.135	82.9582	91.40911

Average		69.28276	73.34964	70.94193	53.16378	60.77463	82.86249

**Table 6:** Average performance analysis of classifiers in terms of parameters with average to six optimization techniques for different gene selection methods using 30-60-90 selected genes.

Classifiers	Gene selection	Parameters (%)			
		Accuracy	Perfect Classification	Performance Index	GDR
RF	30	83.59433	67.18491	45.30313	66.88702
	60	87.60384	75.02862	62.93849	74.75641
	90	82.42909	64.85208	40.91995	64.85275

Adaboost	30	83.87583	67.49332	46.09174	65.12554
	60	87.02927	74.44755	59.07174	67.71936
	90	83.56061	67.12004	45.80861	59.94513

LR	30	85.74922	71.49516	54.40771	59.299
	60	84.01784	68.03036	44.20435	46.31734
	90	88.0159	76.02367	65.72766	67.39558

DT	30	89.02009	78.04018	64.62035	71.93313
	60	88.21978	76.84355	64.60034	71.21466
	90	87.8683	75.7366	61.76893	75.73805

QDA	30	87.4349	74.86979	58.91599	74.53245
	60	88.93083	78.09815	67.8271	77.85126
	90	91.22996	82.36952	72.44417	82.37038

## Data Availability

The data availability will be provided to the genuine researchers upon verifiability and request to the corresponding author.
